# Intrauterine diabetic milieu instigates dysregulated adipocytokines production in F1 offspring

**DOI:** 10.1186/s40781-016-0125-1

**Published:** 2017-01-09

**Authors:** Shady H. Tawfik, Maha M. Haiba, Mohamed I. Saad, Taha M. Abdelkhalek, Mervat Y. Hanafi, Maher A. Kamel

**Affiliations:** 1 Department of Biochemistry, Medical Research Institute, Alexandria University, P.O. Box 21561, 165 Elhorreya Avenue, Alexandria, Egypt; 2 Department of Human Genetics, Medical Research Institute, Alexandria University, Alexandria, Egypt

**Keywords:** Diabetes, Insulin resistance, Adipokines, Fatal origin of disease

## Abstract

**Background:**

Intrauterine environment plays a pivotal role in the origin of fatal diseases such as the metabolic syndrome. Diabetes is associated with low-grade inflammatory state and dysregulated adipokines production. The aim of this study is to investigate the effect of maternal diabetes on adipocytokines (adiponectin, leptin and TNF-α) production in F1 offspring in rats.

**Methods:**

The offspring groups were as follows: F1 offspring of control mothers under control diet (CD) (**CF1-CD**), F1 offspring of control mothers under high caloric diet (HCD) (**CF1-HCD**), F1 offspring of diabetic mothers under CD (**DF1-CD**), and F1 offspring of diabetic mothers under HCD (**DF1-HCD**). Every 5 weeks post-natal, 10 pups of each subgroup were culled to obtain blood samples for biochemical analysis.

**Results:**

The results indicate that DF1-CD and DF1-HCD groups exhibited hyperinsulinemia, dyslipidemia, insulin resistance and impaired glucose homeostasis compared to CF1-CD (*p > 0.05*). DF1-CD and DF1-HCD groups had high hepatic and muscular depositions of TGs. The significant elevated NEFA level only appeared in offspring of diabetic mothers that was fed HCD. DF1-CD and DF1-HCD groups demonstrated low serum levels of adiponectin, high levels of leptin, and elevated levels of TNF-α compared to CF1-CD (*p > 0.05*). These results reveal the disturbed metabolic lipid profile of offspring of diabetic mothers and could guide further characterization of the mechanisms involved.

**Conclusion:**

Dysregulated adipocytokines production could be a possible mechanism for the transgenerational transmittance of diabetes, especially following a postnatal diabetogenic environment. Moreover, the exacerbating effects of postnatal HCD on NEFA in rats might be prone to adipcytokine dysregulation. Furthermore, dysregulation of serum adipokines is a prevalent consequence of maternal diabetes and could guide further investigations to predict the development of metabolic disturbances.

## Background

There is a compelling clinical and epidemiological evidence suggests that prenatal environment plays a pivotal role in the origin of fatal diseases such as the metabolic syndrome and its components: hypertension, insulin resistance, and dyslipidemia [1]. The altered maternal metabolism in diabetic mothers appears to be associated with a diabetogenic effect in the adult offspring, through adaptations during intrauterine fetal development [2]. This intrauterine programming could occur at the gene, cellular, tissue, organ, and system levels, culminating in long-lasting structural and functional changes [3].

Adiponectin have insulin-sensitizing, anti-diabetic, antioxidant, anti-inflammatory and anti-atherogenic properties through its action on adiponectin receptors, e.g., AdipoR1 and AdipoR2 [4]. Leptin is an adipokines involved in the pathogenesis of obesity, insulin resistance, inflammation, and diabetes. It has a regulatory action on satiety and energy homeostasis, production of inflammatory mediators, and on lipid and carbohydrate metabolism [4]. Diabetes is associated with decreased levels of adiponectin and increased levels of leptin. This reflects a state of adiponectin deficiency and leptin resistance, which could be a possible mechanism for development of diabetes complications [4–6].

Low-grade inflammation is a hallmark of type 2 diabetes and metabolic syndrome [4]. Tumor necrosis factor-α (TNF-α) is an inflammatory cytokine that has a myriad of functions such as mediating apoptosis and regulation of immune system. TNF-α exerts its effects among various cells via its action on its TNFR1 and TNFR2 receptors [4]. Serum TNF-α level is elevated in type 1 and type 2 diabetic patients and is correlated with various complications of diabetes [7, 8].

Intrauterine epigenetic modifications, deterioration of glucose tolerance and oxidative stress could be a possible mechanisms for the transgenerational transmittance of diabetes [9, 10]; however, the exact mechanism is not fully understood. Therefore, the aim of this study is to investigate the effect of maternal diabetes on adipocytokines (adiponectin, leptin and TNF-α) production in F1 offspring in rats, as a potential mechanism for the transgenerational effect of maternal diabetes.

## Methods

### Animals

The study was done in accordance with the ethical guidelines of the Medical Research Institute, Alexandria University, Egypt. Wistar rats were housed 4 per cage at an ambient temperature of 23 ± 1 °C with 12/12 h light/dark cycles and 45 ± 5% humidity.

### Experimental design

Female rats were allocated randomly in two groups: control and diabetic. Diabetes was instigated via neonatal injection of streptozotocin (100 mg/kg) at the day 5 postnatally. After 12 weeks, chronic hyperglycemia was confirmed; where rats exhibited fasting blood glucose (FBG) level >200 mg/dl were considered diabetic. 78 hyperglycemic female rats were obtained and maintained under normal control diet (CD) throughout the experiment.

Pregnancy was established by overnight mating the females (control and diabetic) with normal healthy males. Pregnancy was confirmed next morning by the presence of vaginal mucus plug. Pregnancies were completed to term. After delivery, the offspring were weaned to control diet (CD) or high-caloric diet (HCD) and followed up for 30 weeks. Therefore, the offspring groups were as follows: F1 offspring of control mothers under CD (CF1-CD), F1 offspring of control mothers under HCD (CF1-HCD), F1 offspring of diabetic mothers under CD (DF1-CD), and F1 offspring of diabetic mothers under HCD (DF1-HCD). Pregnancy outcome and composition of diets used were previously published [11]. Every 5 weeks post-natal, 10 pups of each subgroup were culled after overnight fasting to obtain blood samples for biochemical analysis.

### Biochemical analysis

FBG level was measured using Glucometer (One Touch, Johnson and Johnson Co.). Lipid profile was assessed by using a commercial diagnostic kit (Randox (UK)) according to the manufacturer instructions.

### ELISA measurements

Plasma insulin was assayed using ELISA kit (Mercodia). Serum levels of adiponectin, leptin, NEFA and TNF-α were assessed using ELISA kits (Chemicon, RayBio, MyBioSource and R&D Systems respectively) according to the manufacturer instructions.

### Statistical analysis

All statistical analyses were performed using SPSS statistical software version 18 (SPSS, Chicago, IL). The data were analyzed using the one-way analysis of variance (ANOVA) followed by LSD test to compare the mean values from the offspring of diabetic mothers and the offspring of control mothers. *t test* was employed to compare the mean values of females and those of males of the same group at the same age. The results are presented as mean ± SEM or mean ± SD and values of *p > 0.05* were considered non-significantly different, while those of *p < 0.05* were considered significant.

## Results

### Glucose homeostasis parameters

#### Fasting Blood Glucose (FBG) level

**Fig. 1:**
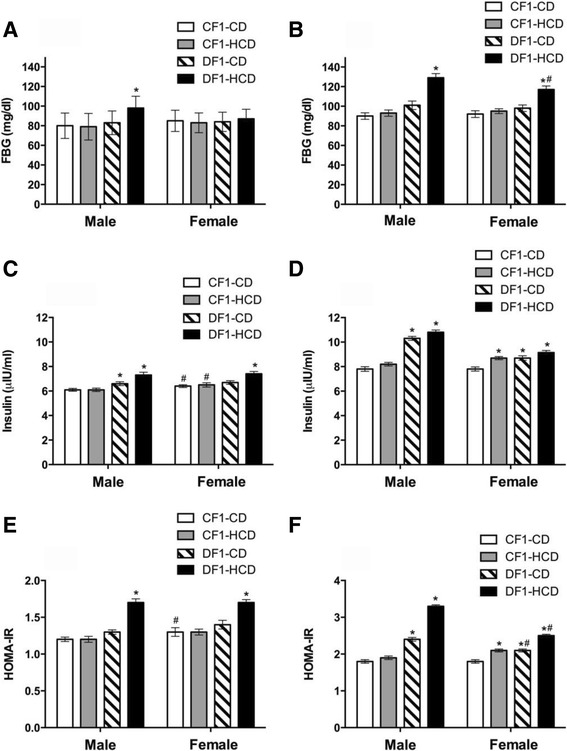
Glucose homeostasis parameters for all study groups at the 5^th^ week (**a**, **c** and **e**) and the 30^th^ week (**b**, **d** and **f**). Data are presented as mean ± SEM (*n* = 10). Abbreviations denote: CF1-CD; F1 offspring of control mothers under CD, CF1-HCD; F1 offspring of control mothers under HCD, DF1-CD; F1 offspring of diabetic mothers under CD, and DF1-HCD; F1 offspring of diabetic mothers under HCD. *Significant different compared to the CF1-CD group by ANOVA (*p < 0.05*). ^#^Significant different from the males of the same group at the same age by *t test* (*p < 0.05*)

At week 5, only males of DF1-HCD showed significantly higher FBG than CF1-CD group. At week 30, males and females of DF1-HCD exhibited significantly higher FBG than CF1-CD group. Furthermore, males of DF1-HCD had significantly higher FBG than females of the same group at the 30^th^ week (Fig. 1).

#### Serum insulin level

At week 5, males of both DF1-CD and DF1-HCD groups and females of DF1-HCD group showed significantly higher insulin levels than CF1-CD group. Also, females of CF1-CD and CF1-HCD groups showed higher insulin levels than their male counterparts (Fig. 1). At week 30, only females of CF1-HCD group as well as all members of DF1-CD and DF1-HCD groups exhibited significantly higher insulin levels than CF1-CD group (Fig. 1).

#### Homeostasis Model Assessment of Insulin Resistance (HOMA-IR)

The insulin resistance index calculated by the HOMA model (HOMA-IR) using fasting serum levels of insulin (μIU/ml) and glucose levels (mmol/l) indicated that males and females of DF1-HCD group were insulin resistant compared to CF1-CD group (Fig. 1). At the 30^th^ week, only females of CF1-HCD group as well as all members of DF1-CD and DF1-HCD groups were insulin resistant compared to CF1-CD group. Moreover, males of DF1-CD and DF1-HCD groups had higher HOMA-IR levels than their female counterparts (Fig. 1).

### Lipid profile

**Fig. 2:**
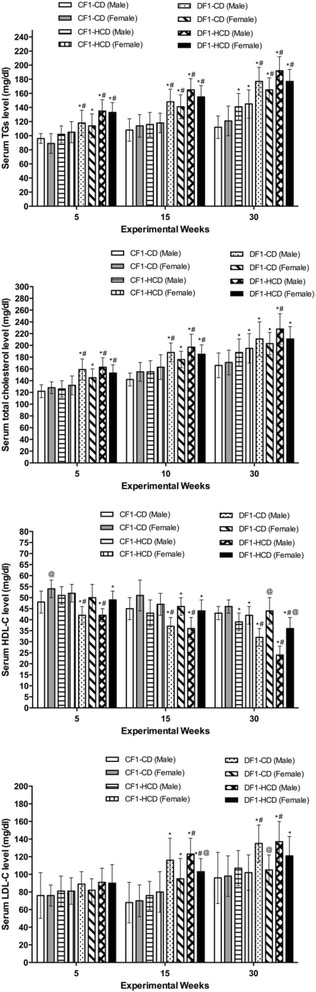
Lipid profile parameters for all study groups. Data are presented as mean ± SD (*n* = 10). Abbreviations denote: CF1-CD; F1 offspring of control mothers under CD, CF1-HCD; F1 offspring of control mothers under HCD, DF1-CD; F1 offspring of diabetic mothers under CD, and DF1-HCD; F1 offspring of diabetic mothers under HCD. *Significant different compared to the CF1-CD group by ANOVA (*p < 0.05*). ^#^Significant different compared to the CF1-CD group by ANOVA (*p < 0.05*). ^@^Significant different from the males of the same group at the same age by *t test* (*p < 0.05*)

All diabetic groups showed significantly elevated serum triglycerides (TGs) level compared to CF1-CD and CF1-HCD groups throughout the 3 time points, except the females of DF1-CD at the 5^th^ week, when they showed only significant difference with CF1-CD. Moreover, CF1-HCD group exhibited a significant high TGs level compared to CF1-CD at the 30^th^ week (Fig. 2).

**Fig. 3:**
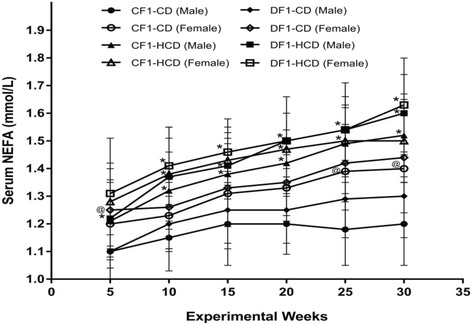
Data are presented as mean ± SD (*n* = 10). Abbreviations denote: CF1-CD; F1 offspring of control mothers under control diet, CF1-HCD; F1 offspring of control mothers under HCD, DF1-CD; F1 offspring of diabetic mothers under control diet, and DF1-HCD; F1 offspring of diabetic mothers under HCD. (*) Significant different from CF1-CD by ANOVA (*p* < 0.05), (#) significant different from CF-HCD by ANOVA (*p* < 0.05), and (@) significant different from male at each age by *t*-test (*p* < 0.05)

Members of CF1-HCD had a significant high NEFA level from week 10 to week 30 compared to CF1-CD, except the females at weeks 15, 25 and 30. All members of DF1-HCD exhibited significantly higher NEFA level than CF1-CD throughout the experiment, except females at the 5^th^ week. Furthermore, there was a significant rise in NEFA level in females of CF1-CD at the 25^th^ and 30^th^ weeks as well as females of DF1-CD at the 5^th^ week, compared to their male counterparts (Fig. 3).

**Fig. 4:**
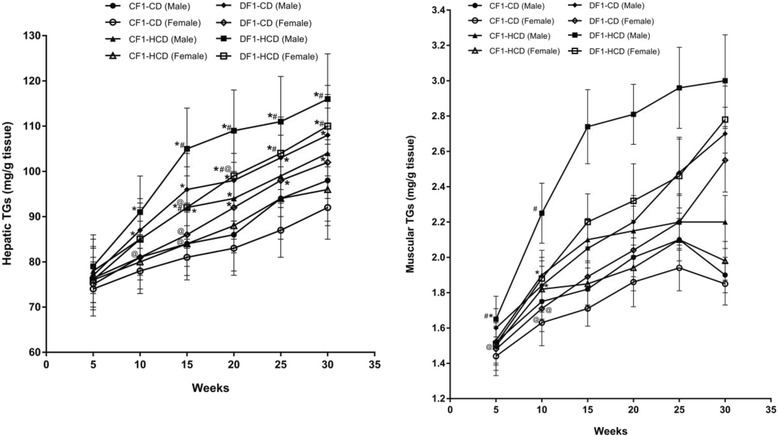
Data are presented as mean ± SD (*n* = 10). Abbreviations denote: CF1-CD; F1 offspring of control mothers under control diet, CF1-HCD; F1 offspring of control mothers under HCD, DF1-CD; F1 offspring of diabetic mothers under control diet, and DF1-HCD; F1 offspring of diabetic mothers under HCD. (*) Significant Different from CF1-CD by ANOVA (*p* < 0.05), (#) significant Different from CF-HCD by ANOVA (*p* < 0.05), and (@) significant different from male at each age by *t*-test (*p* < 0.05)

Members of DF1-CD showed significantly higher hepatic TGs content compared to CF1-CD from the 15^th^ week to the 30^th^ week, except females at the 15^th^ week. Also, all members of DF1-HCD showed a significant rise in their hepatic TGs content from the 10^th^ week compared to CF1-CD, and from the 15^th^ week compared to CF1-HCD, till the end of the experiment (Fig. 4).

CF1-HCD group showed significantly elevated muscular TGs content from the 10^th^ week till the 30^th^ week compared to CF1-CD, except males at week 25, and females at weeks 20 and 30. Also, DF1-CD group exhibited a significant high muscular TGs content at the 15^th^ week till the 30^th^ week compared to CF1-CD. All members of DF1-HCD, except females at the 5^th^ week, had the same pattern throughout the study period. Males of DF1-CD at the 25^th^ and 30^th^ weeks as well as females at the 30^th^ week had a significant higher muscular TGs content than CF1-HCD. All members of DF1-HCD (except females at the 5^th^ week) had a significant elevated muscular TGs content compared to CF1-HCD. Furthermore, females of DF1-CD (except at the 30^th^ week) and DF1-HCD had significantly lower muscular TGs content than their male counterparts (Fig. 4, Tables 1 and 2).

**Table 1:** Hepatic and muscular content of TGs (mg/g tissue) in different study groups

Age (Weeks)	Sex	CF1-CD	CF1-HCD	DF1-CD	DF1-HCD
Hepatic TGs content (mg/g tissue)					
5	Male	76.4 ± 7	78 ± 8	77 ± 6	79 ± 6
	Female	74 ± 6	76 ± 5	75 ± 5	76 ± 5
10	Male	81 ± 8	85 ± 8	87 ± 7	91 ± 8*
	Female	78 ± 5	80 ± 6	81 ± 5***	85 ± 7*
15	Male	84 ± 7	92 ± 9*	96 ± 8*	105 ± 9*,**
	Female	81 ± 5	84 ± 7***	86 ± 7***	92 ± 7*,**,***
20	Male	86 ± 8	94 ± 8*	98 ± 6*	109 ± 9*,**
	Female	83 ± 6	88 ± 6	92 ± 7*	99 ± 10*,**,***
25	Male	94 ± 9	99 ± 9	103 ± 9*	111 ± 10*,**
	Female	87 ± 6	94 ± 7	98 ± 6*	104 ± 8*,**
30	Male	98 ± 9	104 ± 10	108 ± 9*	116 ± 10*,**
	Female	92 ± 7	96 ± 8	102 ± 8*	110 ± 9*,**
Muscular TGs content (mg/g tissue)					
5	Male	1.52 ± 0.12	1.53 ± 0.1	1.6 ± 0.11	1.65 ± 0.13*,**
	Female	1.44 ± 0.11	1.5 ± 0.11	1.48 ± 0.12***	1.51 ± 0.12***
10	Male	1.75 ± 0.1	1.9 ± 0.14*	1.84 ± 0.14	2.25 ± 0.17*,**
	Female	1.63 ± 0.13***	1.82 ± 0.13*	1.71 ± 0.13***	1.88 ± 0.12*,***
15	Male	1.82 ± 0.12	2.1 ± 0.12*	2.05 ± 0.16*	2.74 ± 0.21*,**
	Female	1.71 ± 0.1***	1.85 ± 0.12*,***	1.89 ± 0.17*,***	2.2 ± 0.16*,**,***
20	Male	2 ± 0.11	2.15 ± 0.14*	2.20 ± 0.15*	2.81 ± 0.17*,**
	Female	1.86 ± 0.14***	1.94 ± 0.13***	2.04 ± 0.17*,***	2.32 ± 0.21*,**,***
25	Male	2.1 ± 0.12	2.2 ± 0.15	2.48 ± 0.2*,**	2.96 ± 0.23*,**
	Female	1.94 ± 0.13***	2.1 ± 0.12*	2.2 ± 0.16^*,***^	2.46 ± 0.21*,**,***
30	Male	1.9 ± 0.1	2.2 ± 0.15*	2.7 ± 0.15*,**	3 ± 0.26*,**
	Female	1.85 ± 0.12	1.98 ± 0.11***	2.55 ± 0.18*,**	2.78 ± 0.19*,**,***

Data are presented as mean ± SD (*n* = 10)

Abbreviations denote: *CF1-CD*; F1 offspring of control mothers under control diet, *CF1-HCD*; F1 offspring of control mothers under HCD, *DF1-CD*; F1 offspring of diabetic mothers under control diet, and *DF1-HCD*; F1 offspring of diabetic mothers under HCD

*Significant Different from CF1-CD by ANOVA (*p < 0.05*)

**Significant Different from CF-HCD by ANOVA (*p < 0.05*)

***Significant different from male at each age by *t-test (p < 0.05)*

**Table 2:** Serum NEFA level (mmol/ml) in different study groups

Age (Weeks)	Sex	CF1-CD	CF1-HCD	DF1-CD	DF1-HCD
5	Male	1.1 ± 0.10	1.21 ± 0.14	1.1 ± 0.10	1.22 ± 0.10*
	Female	1.2 ± 0.16	1.28 ± 0.12	1.25 ± 0.17***	1.31 ± 0.20
10	Male	1.15 ± 0.12	1.32 ± 0.14*	1.2 ± 0.10	1.37 ± 0.14*
	Female	1.23 ± 0.12	1.38 ± 0.17*	1.26 ± 0.15	1.41 ± 0.14*
15	Male	1.20 ± 0.15	1.38 ± 0.13	1.25 ± 0.14	1.41 ± 0.12*
	Female	1.31 ± 0.18	1.43 ± 0.12	1.33 ± 0.14	1.46 ± 0.12*
20	Male	1.20 ± 0.11	1.42 ± 0.12*	1.25 ± 0.12	1.5 ± 0.10*
	Female	1.33 ± 0.12	1.47 ± 0.13*	1.35 ± 0.12	1.5 ± 0.16*
25	Male	1.18 ± 0.13	1.49 ± 0.14*	1.29 ± 0.14	1.54 ± 0.12*
	Female	1.39 ± 0.14***	1.5 ± 0.12	1.42 ± 0.14	1.54 ± 0.17*
30	Male	1.20 ± 0.15	1.52 ± 0.15*	1.3 ± 0.15	1.6 ± 0.14*
	Female	1.4 ± 0.20***	1.5 ± 0.15	1.44 ± 0.20	1.63 ± 0.17*

Data are presented as mean ± SD (*n* = 10)

Abbreviations denote: *CF1-CD*; F1 offspring of control mothers under control diet, *CF1-HCD*; F1 offspring of control mothers under HCD, *DF1-CD*; F1 offspring of diabetic mothers under control diet, and *DF1-HCD*; F1 offspring of diabetic mothers under HCD

*Significant different from CF1-CD by ANOVA (*p < 0.05*)

***Significant different from male at each age by *t-test (p < 0.05)*

All diabetic groups exhibited elevated serum total cholesterol level compared to CF1-CD throughout the 3 time points. CF1-HCD group had higher serum total cholesterol level than CF1-CD only at the 30^th^ week. Furthermore, males of DF1-CD showed higher serum total cholesterol level compared to CF1-HCD at the 5^th^ and 30^th^ weeks. All DF1-HCD members had a significant increase in their serum total cholesterol level compared to CF1-HCD throughout the follow-up time points, except the females at week 30, which did not show this significant difference (Fig. 2).

Regarding serum high-density lipoprotein-cholesterol (HDL-C) levels, all diabetic groups, except females of DF1-CD at the 5^th^ and 30^th^ weeks, showed lower HDL-C levels than CF1-CD group. Also, all males of the diabetic groups at the 3 tome points as well as females of DF1-HCD at the 30^th^ week showed significantly lower HDL-C level compared to CF1-HCD. Furthermore, members of CF1-HCD at the 30^th^ week exhibited a significant lower HDL-C than CF1-CD group. Females had higher HDL-C level throughout the experiments; however, only CF1-CD at the 5^th^ week as well as diabetic groups at the 30^th^ week showed a significant sex difference (Fig. 2).

All diabetic groups at the 15^th^ and 30^th^ weeks, except females of DF1-CD at the 30^th^ week, showed high levels of low-density lipoprotein-cholesterol levels (LDL-C) compared to CF1-CD group. Moreover, all members of DF1-HCD at the 15^th^ week as well as males of diabetic groups at the 30^th^ week showed a significant elevated LDL-C level than CF1-HCD group. Significant sex difference appeared only in DF1-HCD at the 15^th^ week and in DF1-CD at the 30^th^ week (Fig. 2).

### Serum levels of cytokines

**Fig. 5:**
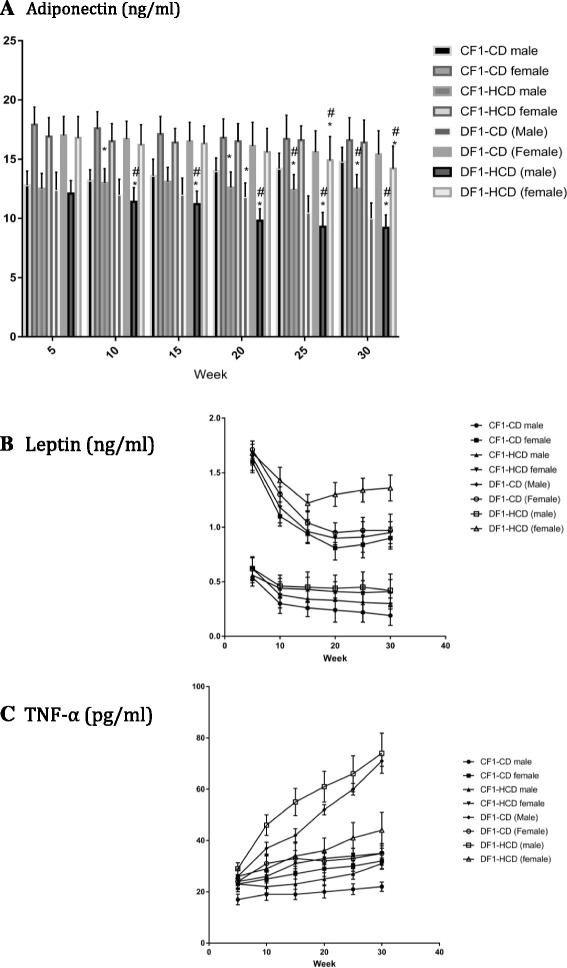
Data are presented as mean ± SD (*n* = 10). Abbreviations denote: CF1-CD; F1 offspring of control mothers under control diet, CF1-HCD; F1 offspring of control mothers under HCD, DF1-CD; F1 offspring of diabetic mothers under control diet, and DF1-HCD; F1 offspring of diabetic mothers under HCD. (*) Significant different from CF1-CD by ANOVA (*p* < 0.05), (#) significant different from CF-HCD by ANOVA (*p* < 0.05), and (@) significant different from male at each age by *t*-test (*p* < 0.05)

Females had significant higher serum adiponectin levels than their male counterparts in all study groups (except DF1-CD at the 10^th^ week and DF1-HCD at the 25^th^ week) throughout the study period. Males of CF1-HCD and DF1-CD showed significantly lower serum adiponectin levels than CF1-CD from the 20^th^ week to the 30^th^ week. Males of DF1-HCD (from weeks 10 till 30) and females of the same group (at the 25^th^ and 30^th^ weeks) exhibited significantly lower serum adiponectin levels than CF1-CD. Males of DF1-CD (at weeks 25 and 30), males of DF1-HCD (at all study period except the 5^th^ week) and females of DF1-HCD (at weeks 25 and 30) had a significantly lower adiponectin levels compared to CF1-HCD (Fig. 5).

Females had significant elevated serum leptin levels than their male counterparts in all study groups throughout the study period. Throughout the study, males of CF1-HCD (except the 5^th^ week), all members of DF1-CD (except females at week 5 and 30) and all members of DF1-HCD (except females at week 5) had a significantly lower leptin levels compared to CF1-CD. Males of DF1-CD (except at weeks 5 and 10) had significantly lower leptin levels than CF1-HCD, while females of the same group at weeks 5 and 10 had significantly higher leptin levels compared to CF1-HCD. Females of DF1-HCD (except at week 5) showed significantly higher leptin levels compared to CF1-HCD (Fig. 5).

Females had significant higher serum TNF-α levels than their male counterparts in all study groups (except CF1-HCD and DF1-CD at the 5^th^ week) throughout the study period. All members of DF1-HCD and all males of DF1-CD and CF1-HCD had significantly higher serum TNF-α levels compared to CF1-CD. Also, females of CF1-HCD (at weeks 15, 20, 25) and females of DF1-CD (at weeks 10 and 15) showed significantly higher serum TNF-α levels compared to CF1-CD. Moreover, all males of DF1-CD at every time point and females of the same group at week 10 exhibited significantly higher serum TNF-α levels compared to CF1-HCD. All males of DF1-HCD at every time point and females of the same group at weeks 10,25 and 30 exhibited significantly higher serum TNF-α levels compared to CF1-HCD (Fig. 5, and Table 3).

**Table 3:** Serum levels of cytokines in different study groups

Age (Weeks)	Sex	CF1-CD	CF1-HCD	DF1-CD	DF1-HCD
Adiponectin (ng/ml)					
5	Male	12.8 ± 1.2	12.5 ± 1.3	12.4 ± 1.5	12.1 ± 1.1
	Female	17.9 ± 1.5***	16.9 ± 1.6***	17.0 ± 1.6***	16.8 ± 1.8***
10	Male	13.2 ± 0.9	13 ± 1.2	12.0 ± 1.3*	11.4 ± 1.2*,**
	Female	17.6 ± 1.4***	16.5 ± 1.5***	16.7 ± 1.5	16.2 ± 1.7***
15	Male	13.6 ± 1.4	13.1 ± 1.2	12.0 ± 1.4	11.2 ± 1.1*,**
	Female	17.1 ± 1.5***	16.4 ± 1.2***	16.5 ± 1.6***	16.3 ± 1.5***
20	Male	14 ± 1.1	12.6 ± 1.32*	11.8 ± 1.2*	9.8 ± 1*,**
	Female	16.8 ± 1.6***	16.5 ± 1.5***	16.1 ± 2***	15.6 ± 2***
25	Male	14.2 ± 1.3	12.4 ± 1.3*	10.5 ± 1.4*,**	9.3 ± 1.2*,**
	Female	16.7 ± 2***	16.6 ± 2***	15.6 ± 1.8***	14.9 ± 2*,**
30	Male	14.8 ± 1.2	12.5 ± 1.2*	10.0 ± 1.3*,**	9.2 ± 1.1*,**
	Female	16.6 ± 1.9***	16.4 ± 1.9***	15.4 ± 2***	14.2 ± 1.9^*,**,***^
Leptin (ng/ml)					
5	Male	0.54 ± 0.08	0.56 ± 0.07	0.62 ± 0.11*	0.62 ± 0.1*
	Female	1.6 ± 0.1***	1.64 ± 0.12***	1.71 ± 0.08***,**	1.68 ± 0.11^**^
10	Male	0.3 ± 0.09	0.44 ± 0.09*	0.38 ± 0.12*	0.46 ± 0.1*
	Female	1.1 ± 0.09***	1.18 ± 0.14***	1.3 ± 0.07*,**,***	1.43 ± 0.12*,**,***
15	Male	0.26 ± 0.08	0.43 ± 0.11*	0.34 ± 0.09*,**	0.45 ± 0.14*
	Female	0.94 ± 0.08***	0.96 ± 0.1***	1.04 ± 0.11***	1.22 ± 0.08*,**,***
20	Male	0.24 ± 0.11	0.41 ± 0.09*	0.330.08*,**	0.44 ± 0.12*
	Female	0.81 ± 0.11***	0.9 ± 0.06***	0.95 ± 0.09*,***	1.3 ± 0.11*,**,***
25	Male	0.22 ± 0.09	0.4 ± 0.11*	0.31 ± 0.1*,**	0.45 ± 0.14*
	Female	0.84 ± 0.12***	0.91 ± 0.14***	0.97 ± 0.12*,***	1.34 ± 0.1*,**,***
30	Male	0.19 ± 0.09	0.41 ± 0.11*	0.3 ± 0.05*,**	0.42 ± 0.15*
	Female	0.9 ± 0.1***	0.95 ± 0.1***	0.97 ± 0.15***	1.36 ± 0.12*,**,***
TNF-α (pg/ml)					
5	Male	17 ± 2.1	23 ± 2.1*	26 ± 2.4*,**	29 ± 2.3*,**
	Female	23 ± 3***	24 ± 2.5	24 ± 2.7	26 ± 3*,***
10	Male	19 ± 2.3	22 ± 2.4*	37 ± 2.3*,**	46 ± 4*,**
	Female	25 ± 3***	26 ± 2.6***	31 ± 3.4*,**,***	29 ± 5*,**,***
15	Male	19 ± 2	23 ± 2*	42 ± 2.6*,**	55 ± 5.3*,**
	Female	27 ± 4***	31 ± 3*,***	33 ± 3.1*,***	34 ± 5*,***
20	Male	20 ± 2.4	25 ± 2.4*	52 ± 2*,**	61 ± 6*,**
	Female	29 ± 3***	33 ± 2.5*,***	32 ± 3***	36 ± 5*,***
25	Male	21 ± 2.1	27 ± 2.1*	60 ± 2.3*,**	66 ± 7*,**
	Female	30 ± 2***	34 ± 3*,***	33 ± 4***	41 ± 6*,**,***
30	Male	22 ± 1.8	31 ± 2.2*	71 ± 2.1*,**	74 ± 7.8*,**
	Female	32 ± 3***	35 ± 3***	35 ± 3.8***	44 ± 7*,**,***

Data are presented as mean ± SD (*n* = 10)

Abbreviations denote: *CF1-CD*; F1 offspring of control mothers under control diet, *CF1-HCD*; F1 offspring of control mothers under HCD, *DF1-CD*; F1 offspring of diabetic mothers under control diet, and *DF1-HCD*; F1 offspring of diabetic mothers under HCD

*Significant different from CF1-CD by ANOVA (*p < 0.05*)

**Significant different from CF-HCD by ANOVA (*p < 0.05*)

***Significant different from male at each age by *t-test (p < 0.05)*

## Discussion

According to the hypothesis of fetal origin of adult diseases, the intrauterine environment may have a critical influence on long-term health and disease of the offspring. Therefore, intrauterine milieu may alter the metabolic status of the fetus, driving an insulin resistance state and facilitates the development of type 2 diabetes mellitus and metabolic syndrome in the adult life, especially following a postnatal obesogenic environment [12]. In support of this idea, our findings indicate that F1 offspring of diabetic mothers exhibited impaired glucose homeostasis, dyslipidemia, insulin resistance, and dysregulated adipokines production, paving the way for the development of diabetes and its complications, especially when they encounter diabetogenic environment.

Dyslipidemia is one of the major risk factors for the development of cardiovascular diseases in the context of diabetes mellitus. The cardinal features of diabetic dyslipidemia are: a high plasma TGs level, low HDL-C level and elevated level of small dense LDL-C particles. These lipid changes are attributed to the increased NEFA flux secondary to adipose tissue insulin resistance [13]. The increase in NEFA flux instigates insulin resistance in liver and muscle through direct or indirect (via triglyceride deposits) generation of metabolites, interfering with insulin signalling pathway [14]. Moreover, NEFA play a crucial role in the development of β-cell dysfunction [15].

Hyperinsulinemia has a pivotal role in the development of hepatosteatosis and hepatic insulin resistance [16]. Consistently, offspring of diabetic mothers showed significant elevated TGs content in their livers and muscles. However, the elevated NEFA level only appeared in offspring of diabetic mothers that was fed high caloric diet, suggesting the role of the postnatal environment in the development of metabolic disturbances in the offspring. In human, young, normoglycemic and insulin-resistant offspring of parents with type 2 diabetes showed impaired muscular signaling with enhanced intramyocellular lipid content [17].

Our data indicates that in-utero maternal diabetes is sufficient for alteration the adipocytokines production in the offspring during their adult life, and the diabetogenic environment exaggerates this effect. Offspring of diabetic mothers showed low levels of adiponectin, high levels of leptin, and elevated levels of TNF-α. Assessment of the serum adiponectin in diabetic patients is useful for evaluating the metabolic status and for determining the risk of cardiovascular diseases [18].

It has been shown that inflammatory markers are increased in the offspring of type 2 diabetic patients [19–21]. Also, there is a strong association between markers of inflammatory activity and endothelial dysfunction, the trigger for cardiovascular diseases [4]. Interestingly, increased inflammatory mediators may predict the future development of obesity and diabetes. The elevated concentrations of TNF-α and interleukin-6 (IL-6) might interfere with insulin action by suppressing insulin signal transduction [22]. Therefore, inactivation of TNF-α by infliximab ameliorates diabetic nephropathy in streptozotocin-induced diabetic mice [23].

## Conclusion

In conclusion, dysregulated adipocytokines production could be a possible mechanism for the transgenerational transmittance of diabetes, especially when the offspring encounter diabetogenic environment. Furthermore, serum adipokines level could be a powerful tool to predict the development of metabolic disturbances in offspring.

## References

[1] Gluckman PD, Hanson MA (2004). The developmental origins of the metabolic syndrome. Trends Endocrinol Metab.

[2] Aerts L, Van Assche FA (2006). Animal evidence for the transgenerational development of diabetes mellitus. Int J Biochem Cell Biol.

[3] Fowden AL, Giussani DA, Forhead AJ (2006). Intrauterine programming of physiological systems: causes and consequences. Physiology.

[4] Saad MI, Abdelkhalek TM, Saleh MM, Kamel MA, Youssef M, Tawfik SH, Dominguez H. Insights into the molecular mechanisms of diabetes-induced endothelial dysfunction: focus on oxidative stress and endothelial progenitor cells. Endocrine. 2015;50(537):1–31. doi:10.1007/s12020-015-0709-4. 10.1007/s12020-015-0709-4 26271514

[5] Saad MI, Kamel MA, Hanafi MY. Modulation of adipocytokines production and serum NEFA level by metformin, glimepiride, and sitagliptin in HFD/STZ diabetic rats. Biochem Res Int. 2015. 10.1155/2015/138134 PMC4369950 25838947

[6] Tawfik SH, Mahmoud BF, Saad MI, Shehata M, Kamel MA, Helmy MH. Similar and additive effects of ovariectomy and diabetes on insulin resistance and lipid metabolism. Biochem Res Int. 2015. 10.1155/2015/567945 PMC4365318 25834745

[7] Zorena K, Mysliwska J, Mysliwiec M, Balcerska A, Lipowski P, Raczynska K (2007). Relationship between serum levels of tumor necrosis factor-alpha and interleukin-6 in diabetes mellitus type 1 children. Cen Eur J Immunol.

[8] Makino N, Maeda T, Sugano M, Satoh S, Watanabe R, Abe N (2005). High serum TNF-α level in Type 2 diabetic patients with microangiopathy is associated with eNOS down-regulation and apoptosis in endothelial cells. J Diabetes Complicat.

[9] Liguori A, Puglianiello A, Germani D, Deodati A, Peschiaroli E, Cianfarani S. Epigenetic changes predisposing to type 2 diabetes in intrauterine growth retardation. Front Endocrinol. 2010;1 10.3389/fendo.2010.00005 PMC3355861 22649352

[10] Reusens B, Remacle C (2006). Programming of the endocrine pancreas by the early nutritional environment. Int J Biochem Cell Biol.

[11] Kamel MA (2012). Prenatal effects of different intra-uterine milieus on fetal glucose sensing mechanisms during gestation in rats.

[12] Kanaka‐Gantenbein C (2010). Fetal origins of adult diabetes. Ann N Y Acad Sci.

[13] Mooradian AD (2009). Dyslipidemia in type 2 diabetes mellitus. Nat Clin Pract Endocrinol Metab.

[14] Delarue J, Magnan C (2007). Free fatty acids and insulin resistance. Curr Opin Clin Nutr Metab Care.

[15] Boden G, Shulman G (2002). Free fatty acids in obesity and type 2 diabetes: defining their role in the development of insulin resistance and β‐cell dysfunction. Eur J Clin Investig.

[16] Steneberg P, Sykaras AG, Backlund F, Straseviciene J, Söderström I, Edlund H (2015). Hyperinsulinemia enhances hepatic expression of the fatty acid transporter Cd36 and provokes hepatosteatosis and hepatic insulin resistance. J Biol Chem.

[17] Morino K, Petersen KF, Dufour S, Befroy D, Frattini J, Shatzkes N, Neschen S, White MF, Bilz S, Sono S (2005). Reduced mitochondrial density and increased IRS-1 serine phosphorylation in muscle of insulin-resistant offspring of type 2 diabetic parents. J Clin Investig.

[18] Hosokawa Y, Yamada Y, Obata Y, Baden MY, Saisho K, Ihara A, Yamamoto K, Katsuragi K, Matsuzawa Y (2011). Clinical significance of serum adiponectin in Japanese diabetic patients. Diabetol Int.

[19] Ruotsalainen E, Vauhkonen I, Salmenniemi U, Pihlajamäki J, Punnonen K, Kainulainen S, Jalkanen S, Salmi M, Laakso M (2008). Markers of endothelial dysfunction and low-grade inflammation are associated in the offspring of type 2 diabetic subjects. Atherosclerosis.

[20] Ruotsalainen E, Salmenniemi U, Vauhkonen I, Pihlajamäki J, Punnonen K, Kainulainen S, Laakso M (2006). Changes in inflammatory cytokines are related to impaired glucose tolerance in offspring of type 2 diabetic subjects. Diabetes Care.

[21] Maltezos E, Papazoglou D, Exiara T, Papazoglou L, Karathanasis E, Christakidis D, Ktenidou-Kartali S (2002). Tumour necrosis factor-α levels in non-diabetic offspring of patients with type 2 diabetes mellitus. J Int Med Res.

[22] Dandona P, Aljada A, Bandyopadhyay A (2004). Inflammation: the link between insulin resistance, obesity and diabetes. Trends Immunol.

[23] Yamakawa I, Kojima H, Terashima T, Katagi M, Oi J, Urabe H, Sanada M, Kawai H, Chan L, Yasuda H (2011). Inactivation of TNF-α ameliorates diabetic neuropathy in mice. Am J Physiol-Endocrinol Metab.

